# Conduction block and thin and hypokinetic myocardial segments in feline hypertrophic cardiomyopathy

**DOI:** 10.17221/14/2025-VETMED

**Published:** 2025-08-30

**Authors:** Yunjin Sung, Jinyeong Park, Yeon Chae, Taesik Yun, Byeong-Teck Kang, Hakhyun Kim

**Affiliations:** Laboratory of Veterinary Internal Medicine, College of Veterinary Medicine, Chungbuk National University, Cheongju, Republic of Korea

**Keywords:** electrocardiogram, feline, fibrosis, myocardial remodelling

## Abstract

A 12-year-old castrated male domestic shorthair cat was referred for respiratory distress. Physical examination revealed a systolic heart murmur at the left apex and crackles in all lung fields. Thoracic radiography showed Valentine-shaped cardiomegaly, pulmonary oedema, and pleural effusion. Echocardiography revealed focal thickening of the interventricular septum [11.01 mm; reference interval (RI) = 3.00–5.20 mm] and left ventricular posterior wall (7.41 mm; RI = 3.00–5.10 mm) during diastole. In the apex region, the free wall was focally thinned to approximately 1.6 mm with hypokinetic myocardial movement, indicating thin and hypokinetic myocardial segments. Additionally, decreased left atrial fractional shortening (12.5%; RI = 23.9–34.9%) and an increased left atrial-to-aortic ratio (2.87; RI = 0.88–1.43) were observed, along with spontaneous echocardiographic contrast in the left atrium, indicating increased thrombotic risk. The electrocardiogram showed a left axis deviation with small R waves and deep S waves in lead II, which is consistent with a left anterior fascicular block caused by delayed conduction in the left anterior fascicle. This case report describes the coexistence of a left anterior fascicular block and thin, hypokinetic myocardial segments in feline hypertrophic cardiomyopathy, suggesting a possible pathophysiological link.

Hypertrophic cardiomyopathy (HCM) is the most prevalent myocardial disorder in cats, characterised by diffuse or regional left ventricular (LV) wall thickening with a non-dilated LV chamber. On echocardiography, HCM is diagnosed when LV wall thickness exceeds 6 mm (Luis Fuentes et al. 2020). However, localised segments of LV wall thinning are occasionally observed in feline HCM. Thin and hypokinetic myocardial segments (THyMS) are characterised by focal myocardial thinning (1.6–1.9 mm) and reduced contractility, often associated with advanced HCM and a poor prognosis (Novo Matos et al. 2023).

In addition to structural abnormalities, conduction disturbances are frequently observed in cats with advanced HCM. The left anterior fascicular block (LAFB) is a common type of conduction block, which results from delayed electrical activation in the left anterior fascicle. On an electrocardiogram (ECG), LAFB is characterised by a marked left axis deviation (LAD) with small Q waves and tall R waves in leads I and aVL and deep S waves in leads II, III, and aVF (Willis et al. 2018).

A human study suggests that cardiac conduction abnormalities correlate with regions of structural myocardial wall thinning (Komatsu et al. 2013). However, the coexistence of conduction abnormalities and focal myocardial thinning has not been reported in cats. This is the first case report to document the coexistence of LAFB and THyMS in feline HCM.

## Case description

Written informed consent was obtained from the cat owner. A 12-year-old castrated male domestic shorthair cat weighing 5.5 kg was referred because of respiratory distress and suspected congestive heart failure (CHF). Upon examination, the cat exhibited tachypnoea with a respiratory rate of 130 breaths per minute and a heart rate of 165 beats per minute. SpO_2_ was 97% with oxygen supplementation. Systolic blood pressure was measured at 96 mmHg using the oscillometric method. Auscultation revealed a regular heart rhythm with a grade 4/6 systolic heart murmur at the left apex, a gallop sound, and crackles throughout all lung fields.

Thoracic radiographs revealed valentine-shaped cardiomegaly with a vertebral heart size of 8.2 [reference interval (RI) = 6.8–7.8 (Ghadiri et al. 2008)]. Alveolar and interstitial patterns were evident in the bilateral caudal lung lobes, consistent with pulmonary oedema. Interlobar fissure lines suggested pleural effusion.

**Figure 1:**
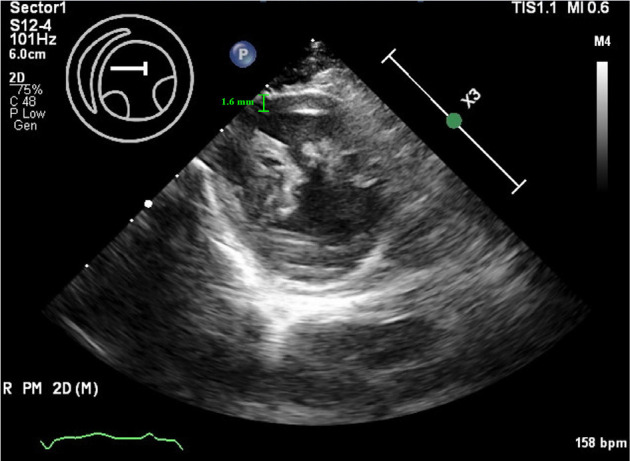
B-mode echocardiographic image showing focal thinning of the left ventricular free wall (green line)

Two-dimensional echocardiography revealed focal thickening of the interventricular septum (maximum diameter, 11.01 mm; RI = 3.00–5.20 mm) and the LV posterior wall (maximum diameter, 7.41 mm; RI = 3.00–5.10 mm) during diastole in the right parasternal long-axis view (Haggstrom et al. 2016). Focal thinning of the LV free wall to approximately 1.6 mm and hypokinesis were observed in the right parasternal short-axis view (Figure 1).

**Figure 2:**
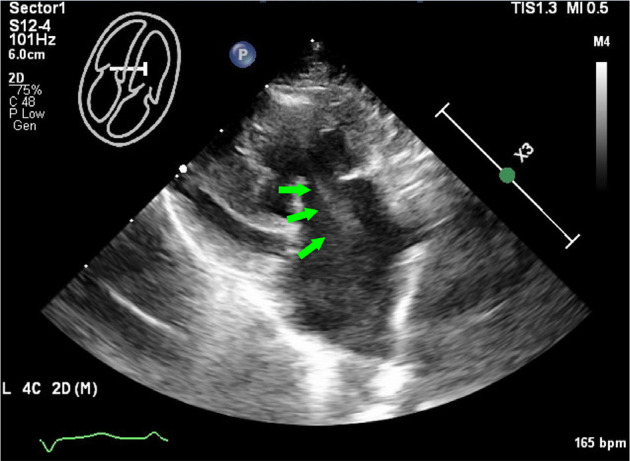
B-mode echocardiographic image showing spontaneous echocardiographic contrast (green arrow) in the left atrium in the left apical four-chamber view

The anterior apex wall was approximately 1.6 mm thick and exhibited hypokinetic myocardial movement, consistent with THyMS, in the right parasternal short-axis view. Spontaneous echocardiographic contrast (SEC) was identified in the left atrium, indicating an increased risk of thrombus formation [Figure 2; a video presented as Electronic Supplementary Material (ESM) S1].

**Figure 3:**
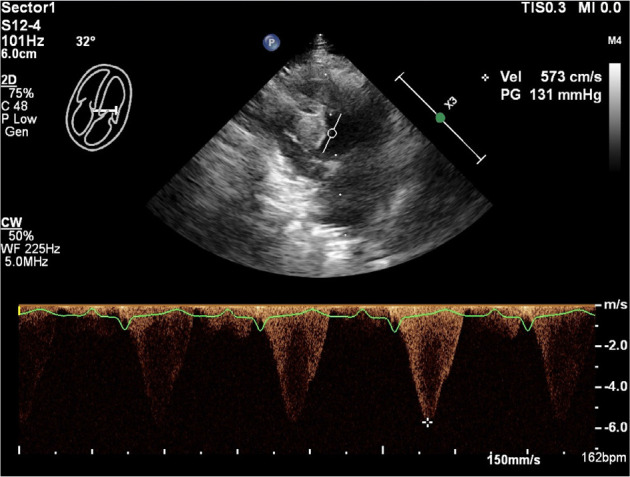
Continuous-wave Doppler echocardiographic images of the left ventricular outflow tract Peak velocity of 5.73 m/s and a pressure gradient of 131 mmHg were measured, consistent with severe dynamic left ventricular outflow tract obstruction

Systolic anterior motion of the mitral valve was present, with severe dynamic LV outflow tract obstruction (peak velocity: 5.73 m/s; pressure gradient: 131 mmHg (Hirose et al. 2024; Figure 3).

The decreased systolic function of the left atrium was evidenced by left atrial (LA)-fractional shortening (FS) of 12.5% [RI = 23.9–34.9% (Machado et al. 2024)], while LV-FS was 29.3% which is near the lower limit of the reference interval (RI = 30–49%). Additional findings included an increased left atrial-to-aortic ratio of 2.87 (RI = 0.88–1.43), indicating moderate-to-severe LA dilation (Haggstrom et al. 2016). The E : A ratio was 1.9 (RI > 1), and the E : E' ratio was 14.1 (RI > 9), both indicative of elevated LV filling pressures consistent with diastolic dysfunction (Schober and Chetboul 2015).

**Figure 4:**
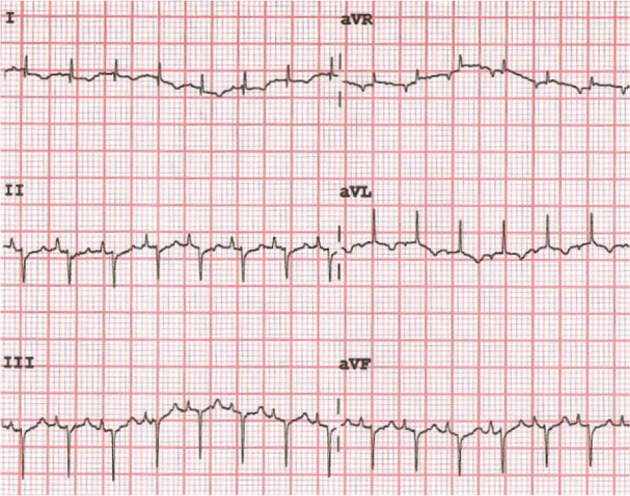
Six-lead electrocardiography recording obtained from the cat diagnosed with hypertrophic cardiomyopathy with concurrent left anterior fascicular block The cat exhibited regular sinus rhythms with a heart rate of 188 beats per minute. Small Q waves and tall R waves were observed in leads I and aVL, while small R waves with deep S waves were present in leads II, III, and aVF. The mean electrical axis was –71°, indicating LAD. Paper speed = 25 mm/s; 10 mm = 1 mV

Electrocardiography was performed to monitor the critically ill cat. A six-lead ECG was recorded with the cat in the right lateral recumbency. The ECG showed a sinus rhythm with a heart rate of 188 beats per minute. The QRS complexes were negative in leads II and III and aVF (small R wave and deep S waves) and positive in leads I and aVL (small Q waves and tall R waves) (Figure 4). The mean electrical axis was –71°, indicating LAD (range, –45° to –90°) (Willis et al. 2018). These findings were consistent with those of LAFB (Willis et al. 2018).

A complete blood count and electrolyte analysis revealed normal results. Serum biochemistry revealed elevated creatinine (229.9 μmol/l; RI = 26.5–185.7 μmol/l). Venous blood gas analysis indicated respiratory alkalosis (pH = 7.42, RI = 7.21–7.41; anion gap = 15.2 mmol/l, RI = 7–16 mmol/l; HCO_3_^–^ = 12.1 mmol/l, RI = 21–28 mmol/l; P_CO2_ = 19.2 mmHg, RI = 28.0–50.0 mmHg). Additionally, thyroid function testing showed a normal total T4 concentration (35.1 nmol/l; RI = 10.3–60.5 nmol/l). Based on these initial tests, the cat was diagnosed with ACVIM stage C HCM with concurrent LAFB and THyMS, contributing to CHF and an increased risk of thromboembolic complications (Luis Fuentes et al. 2020).

Treatment was initiated with oxygen supplementation and three intermittent boluses of 2 mg/kg furosemide (Lasix^®^ inj.; Handok, Eumseong, Republic of Korea) administered intravenously to treat CHF. However, the respiratory rate persisted at 60 breaths/min, indicating that the initial dose was insufficient. To improve pulmonary respiratory distress, a continuous rate infusion of 1 mg/kg/h furosemide was started. Additionally, 0.2 mg/kg of pimobendan (Vetmedin^®^ inj.; LABIANA Life Sciences S.A., Barcelona, Spain) intravenously administered q12h and 150 U/kg of dalteparin (Fragmin^®^ inj.; Pfizer, Puurs, Belgium) administered subcutaneously q8h were administered to improve systolic LA function and prevent thromboembolism, respectively (Kittleson and Cote 2021).

**Figure 5:**
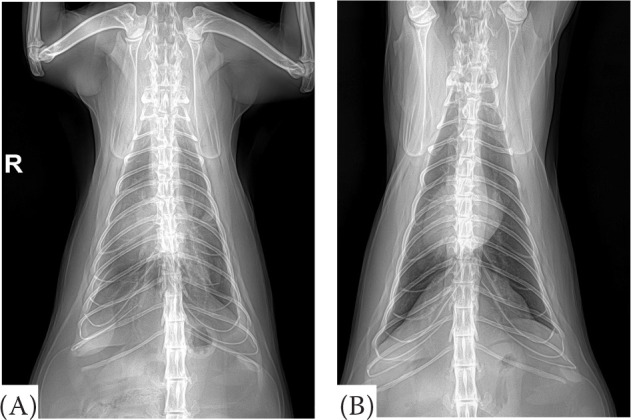
Ventrodorsal thoracic radiographs before (A) and after (B) treatment (A) Initial radiograph shows pulmonary oedema and pleural effusion. (B) A follow-up radiograph taken 2 days after treatment shows improvement in pulmonary oedema and pleural effusion

After 2 days of treatment, respiratory distress was resolved, and the respiratory rate decreased from 60 to 30 breaths per minute. Follow-up thoracic radiographs demonstrated a significant improvement in pulmonary oedema and pleural effusion (Figure 5).

The cat was discharged with a prescription for ongoing oral management, including 0.2 mg/kg of pimobendan (Vetmedin^®^ tab.; LAVET Pharmaceuticals LTD., Budapest, Hungary) q12h, 1.5 mg/kg of furosemide (Lasix^®^ tab.; Handok, Eumseong, Republic of Korea) q12h, 0.5 mg/kg of spironolactone (Aldactone^®^ tab.; Piramal Healthcare, Morpeth, United Kingdom) q12h, 18.75 mg of clopidogrel (Plavix; Shinil Pharm., Chungju, Republic of Korea) q24h, and 1.5 mg/kg of diltiazem (Herben^®^ tab.; HK inno.N, Cheongju, Republic of Korea) q12h. Subsequently, the maintenance dose of furosemide was titrated to maintain a resting or sleeping respiratory rate of <30 breaths/min at home. Over the next 8 months, the cat experienced three additional episodes of CHF, necessitating an increase in furosemide to 2.75 mg/kg q12h. The cat has survived for over 300 days to date without complications.

## DISCUSSION AND CONCLUSIONS

This report describes a cat with HCM showing concurrent LAFB and THyMS. Electrocardiographic identification of LAFB highlighted localised myocardial changes that complemented echocardiographic findings, offering additional insights into structural abnormalities potentially missed on echocardiography.

THyMS is defined as LV segments with end-diastolic wall thickness <3 mm and hypokinesis on echocardiography. THyMS are mainly found in the LV free wall and apex, with histopathology showing severe transmural fibrosis (Novo Matos et al. 2023). The anterior fascicle uses conduction fibres and myocytes to depolarise the antero-superior left ventricle. Myocyte replacement with fibrous tissue impairs conduction and often results in LAFB (Santilli et al. 2018). Correlations between myocardial wall thinning and conduction abnormalities have been demonstrated, with a strong association between regional myocardial wall thinning and low-voltage areas reported in humans (Komatsu et al. 2013). In this case, THyMS were found in the interventricular septum and anterior LV wall (internal and external) with a thickness of 1.6 mm. These changes corresponded closely to areas of conduction disturbance identified on ECG, notably LAFB, suggesting that myocardial thinning and fibrosis disrupt conduction pathways. The concurrent occurrence of THyMS and LAFB suggests a shared pathophysiological mechanism, likely involving fibrosis and remodelling that disrupt conduction.

In this case, echocardiography revealed marked LV hypertrophy with a maximum myocardial wall thickness of 11.01 mm, and SEC in the left atrium, indicating a high thrombotic risk. Decreased LA-FS, indicating left atrial contractile dysfunction, was observed. Additionally, severe LA dilation and diastolic dysfunction were observed. These findings represent critical risk factors for feline HCM, as identified in previous studies (Payne et al. 2015). This cat exhibited multiple risk factors beyond THyMS, suggesting an increased risk of recurrent CHF, consistent with advanced HCM (Kittleson and Cote 2021). Despite poor indicators, including THyMS with a median survival of 153 days (Novo Matos et al. 2023), the cat’s survival exceeded expectations, suggesting that therapeutic combinations, such as pimobendan and diltiazem, may improve survival in advanced HCM.

This report has several limitations. First, histopathological examination of the myocardium was not performed. Histopathological analysis could have provided valuable insights into the potential role of myocardial fibrosis as the underlying pathophysiological link between THyMS and LAFB. Second, follow-up electrocardiographic and echocardiographic evaluations were not conducted. Longitudinal data on THyMS regions could have provided valuable insights into the progression of HCM in this case. However, additional diagnostic evaluations were not pursued at the owner’s request.

In conclusion, this case report describes the coexistence of LAFB and THyMS in a cat with HCM. These findings emphasise the significance of myocardial fibrosis and structural remodelling in advanced diseases and demonstrate the utility of electrocardiography and echocardiography as complementary tools for identifying high-risk cases in feline HCM. The report offers insights for future research on prognostic factors and therapeutic strategies for advanced HCM in cats.
